# A mixed-methods study of the experience of GP educational supervisors

**DOI:** 10.3399/BJGPO.2024.0106

**Published:** 2025-08-13

**Authors:** Derek Elliott, Annabel Shepherd, Karena Anne Hanley, Nitin Gambhir

**Affiliations:** 1 NHS Education Scotland, Glasgow, UK

**Keywords:** General Practice, Clinical Supervision, Medical Education, General practitioners, Primary healthcare

## Abstract

**Background:**

Quality supervision in the clinical learning environment is known to improve future quality of patient care by graduates of that training. The support required by supervisors is not well documented.

**Aim:**

To conduct a needs assessment of educational supervisors (ESs) on the GP training programme.

**Design & setting:**

This was a mixed methods study conducted among the trainer population in the West of Scotland region.

**Method:**

The instrument development mixed methods model was used to design a questionnaire for a whole population survey. These collected quantitative and qualitative data were analysed in a way that triangulated and expanded the data.

**Results:**

One hundred and sixteen ESs (37%) responded and cited time pressures, trainee variation, and professional assessment demands as the biggest challenges to providing quality supervision. Less than half of responders felt they had sufficient time for clinical supervision in the working day. Trainees with additional needs require extra support, a third of ESs did not have sufficient time for pastoral care of their trainee, and the professional assessment burden may have a detrimental effect on the apprenticeship model of GP training. Suggestions for better support were made.

**Conclusion:**

With increasing demands on time, an increased trainer workload and an increase in the number of trainees with more variable needs, the willingness of GPs to become ESs may be reaching a tipping point. This research identified areas for targeting support, but also recommended review of some of the structures of GP training in order to retain quality GP supervision in GP training.

## How this fits in

Educator burnout is increasing more rapidly in general practice training than in hospital specialty training. Educational supervisors (ESs) described their biggest challenges and suggested changes to improve their ability to discharge their role.

## Introduction

GP training practices, compared to non-training practices, significantly improve patient outcomes,^1,2^ through maintaining accreditation, embedding clinical education within the practice, development of leadership qualities, and positive effects on team dynamics.^3^

The quality of the clinical learning environment correlates to later quality of care provided by graduates.^4–7^ A supportive supervisory relationship enhances the knowledge of the trainee,^5,8,9^ their professional development,^7,10,11^ and leads to better retention.^5,10^ Deterrents of quality supervision include a high trainer clinical workload and the time burden of clinical supervision.^5,8,10,11^ Also influential are the availability of medical education for teachers,^7^ the support of the wider organisation,^5,7,11^ and use of the whole clinical team.^10^

The whole time equivalent number of GPs working in Scotland has decreased in the last 10 years,^12^ making the supply of doctors through training more vital. There are around 340 graduates of GP training annually, half of whom are supervised by 311 educational supervisors (ESs) in the West of Scotland region. NHS Education Scotland are responsible for delivery and quality assurance of training. Other influencers on the quality of supervision are the Royal College of General Practitioners and the General Medical Council.^13^

To provide quality supervision, the supervisor must feel supported within the training system. Available literature suggests that most GP trainers get their support principally in their local trainer’s workshops.^3,14,15^ To enhance the Deanery support of trainers, we aimed to understand the experience of being an ES in the West of Scotland and collect actionable support suggestions.

## Method

As a comprehensive needs assessment, we employed a pragmatic philosophy of mixed methods rooted in ’real world’ experience.^16,17^ Both the instrument development model and the triangulation/expansion model were used,^18^ as demonstrated in Supplementary Figure S1. Mixed methods in this context gave a more thorough understanding of complex support needs.

Survey design commenced with the factors that support effective supervision.^5,7,10,11^ With knowledge of these, AS and DE conducted two focus groups of ESs through Trainer’s Group meetings. While a convenience sample, there was also purposeful sampling to include representation from urban, rural, and areas of deprivation. The Forth Valley group comprised 14 participants and the Lanarkshire group 26 participants. The focus groups were audio recorded and anonymised data were transcribed and summarised by AS and DE. They noted four areas of particular relevance to the ESs: workload, supporting the trainee, training assessments, and ES professional development. From the focus group notes, DE devised 6–9 statements for each of these areas. These statements were refined by the study team. The online survey was created using Microsoft Forms, commencing with demographic data. This was followed by the statements for each area, inviting agreement or disagreement along a 5-item Likert scale. Qualitative data were collected on the same form through free-text opportunities. The survey was not piloted but was reviewed by the researchers for face validity.

The survey was promoted in the deanery March training newsletter and emailed to 311 GP ESs on the Trainer register. A follow-up email was sent 4 weeks later and the survey closed after 8 weeks. The research protocol was reviewed by the Scottish Medical Education and Research Consortium, who confirmed that no ethical approval was required. Participation was entirely voluntary and confidential.

The quantitative data were analysed in Microsoft Excel, using simple descriptive statistics and the diverging stacked bar chart function for the Likert data. The qualitative data were analysed using a six step analysis:^19^ familiarisation with the data, open coding, codes organised into categories, categories grouped into themes, themes refined, and representative quotes chosen. Open coding was conducted separately by DE and KH, with 95% congruence of the 14–20 codes for each area. The qualitative data were handled within Microsoft Excel. Data were discussed and reviewed in two further consensus meetings by the authors. Discrepancies were discussed until mutual agreement was reached.

## Results

In total, 116 (37.2%) ESs responded, whose characteristics are shown in Supplementary Table S1. Of the responders, 95% contributed liberally to the free-text responses. The results are reported according to the themes, which dominated the qualitative analysis of the free-text comments, as these four themes explained the data better than the similar (but slightly different) four themes from the focus groups. The quantitative data are also reported.

### Competing time demands

Significant distress was expressed about the toll on training time by increasing clinical demands:

’*The workload is squeezing out the time and energy left for GP training*.’ (GP 89)

Lack of available backfill exacerbates this problem:

’*There are adequate* [educational] *opportunities but finding cover in practice to attend these has been challenging*.’ (GP 96)

In addition, the training workload has increased:

’*It is currently vast and increasing*.’ (GP 32)

Only 6% of responders agreed that the training grant funds sufficient time. Personal time is used by the majority of responders to satisfy training demands. Less than half (49%) of the responders agreed they had enough time for clinical supervision in the working day. A third of trainers reported support of trainees suffers:

’... *Little time in the job to provide pastoral care or document issues*.’ (GP 70)

There were worries about patient safety and medico-legal risk:

’*This is becoming an issue in balancing patient safety/access to facilitating training.*’ (GP 13)

’*Higher concern around litigation and complaints means the support we need to give trainees has risen*.’ (GP 43)

A few responders were coping:

’*Currently the workload is manageable, but I am lucky I have a sturdy practice setup.’* (GP 55)

Some trainers used the wider practice team to help:

’*Patients that can be seen by allied health professionals…. will free up time to supervise/train STs.*’ (GP 6)

Fears for the future were expressed:

’*We are struggling, however, to persuade younger colleagues to take on trainer role so might not be able to continue*.’ (GP 12)

Supplementary Figure S2 shows the quantitative data that demonstrated how the majority of ESs have insufficient protected time and how many find the role to be emotionally draining, and yet the work of training remains satisfying.

There are many calls for improved funding. Assistance with time planning was also suggested, including notice of events:

’*It would be great to get a calendar of events that are happening over a 12-month period*.’ (GP 48)

’[Providing] *a better idea for practices on the amount of time needed by the ES to complete all the necessary requirements for a trainee*.’ (GP 23)

### Trainees have more variable needs

There are more trainees than before and their needs are more variable, with a sense that:

’*Baseline medical knowledge is not as high for many new trainees as in the past*.’ (GP 43)

Trainee attitudes can be problematic:

’*Some trainee’s perception that they are entirely supernumerary is very difficult*.’ (GP 35)

*‘GP registrars are often at stage of life* [family/caring roles/new citizens of Scotland] *where the pathway through training is more complicated*.’ (GP 8)

Trainees experiencing difficulties and international medical graduates require more training investment and have additional needs:

*‘Trainees with additional needs are becoming more common*.’ (GP 11)

The majority of trainers knew how to access help, but find trainees with additional needs challenging:

’*If you have any issues with a trainee this can become intolerably time-consuming and can impact the trainer emotionally*.’ (GP 9)

Unhappiness existed about the competence of a minority of trainees who commence GP training:

’*These trainees are not of the standard foundation training gives UK graduates and it feels like starting with a medical student*.’ (GP 24)

ESs perceived it to be largely their responsibility to get doctors through training:

’*There is far too big an emphasis on trying to get poor and failing trainees to pass through rather than failing them*.’ (GP 38)

Three years of training may be insufficient for some international medical graduates:

’*Often trainees who have never worked in NHS until 1st GPST1 post struggle take longer to complete training*.’ (GP 28)

’*Sometimes if the culture is very different, either they are not receptive to pastoral support or I am not able to give it*.’ (GP 63)

Yet Supplementary Figure S3, which described the quantitative data in this area, demonstrated that the majority of ESs have confidence in their ability to attend to the pastoral and additional development needs of their trainees.

Suggestions included better ’vetting’ of GP trainees, advance notice of trainees with additional needs, and extra support tailored to the requirements of the trainee. That could be financial:

’*With high predictor of selection scoring and outcomes the training grant should be tiered to reflect this*.’ (GP 43)

But it could also be more deanery support:

’*More open dialogue and info about trainees in difficulty would be good*.’ (GP 43)

’*Periodic "check ins" perhaps*.’ (GP 10)

There were suggestions for trainee allocation;

’*Consider the allocation system where the best trainees can choose leafy suburb practices*.’ (GP 53)

for induction;

*‘I think common induction for GPST1s… would be helpful*.’ (GP 18)

and for early human resources support:

’[Human resources] *needs to be better. We had a trainee who had very little support when arrived in country in terms of accommodation, etc*.’ (GP 24)

### Assessment requirements

ESs found:

’[The] *ePortfolio is a huge drain on* [the] *trainer*.’ (GP 35)

Many responders claimed:

’*ePortfolio is exclusively done at home in my own time*.’ (GP 7)

Many comments noted the rising volume of assessments over the last few years. Also frequent changes in assessment requirements have been a source of stress:

’*I find it very difficult to keep up with the constant changes in the requirements for trainees, this is emotionally draining and decreases job satisfaction*!’ (GP 107)

’*A lot of it seems difficult for the trainee to understand how to complete*.’ (GP 63)

Assessment responsibilities may be crowding out important aspects of training:

’*Getting through all this can become the focus for trainee and trainer rather than just learning to do the job well*.’ (GP 66)

Some ESs feel the tension between their role as mentor and assessor:

’*Can be difficult to be brutally honest with trainee*.’ (GP 43)

Many questioned the value of assessments:

*‘I feel the ePortfolio is a series of jumping through ineffectual hoops for the trainee*.’ (GP 94)

Improvement with new technology is noted:

*‘ESRs* [educational supervisor reviews] *are much easier to do on 14fish* [a new IT platform] *than on old system*.’ (GP 114)

Not all responders were unhappy:

’*ePortfolio is a very rigid tool but combined with skills of an ES is a reasonable assessment tool*.’ (GP 59)

There were calls to:

’*Reduce ePortfolio requirements for supervisors.*’ (GP 17)

However, the quantitative data (shown in Supplementary Figure S4) suggest that most trainers were coping with the ePortfolio.

### Support of trainers

Trainers used strategies to self support, such as experience in the role:

’... *is a bit like passing a driving test. There is still a lot of learning on the job*.’ (GP 113)

Another used transferable skills:

’*Feel being a GP is useful in developing skills to provide support to trainees*.’ (GP 30)

Half of trainers found the work to be emotionally draining:

’*We are burning out and the difficulty in fitting in training to a high standard is becoming increasingly challenging*.’ (GP 56)

In contrast, the quantitative data in Supplementary Figure S5 suggest that most ESs feels adequately prepared for their training role, although less than half of them can find protected time for their own professional development.

There were mixed feelings about practice colleagues, some positive and others less positive:

’*I am lucky to work with colleagues who are interested and supportive of GP training*.’ (GP 2)

’*Non-trainers see training activities as skiving often*.’ (GP 7)

Peer support, from trainer’s groups was valued, shown strongly in the quantitative data, Supplementary Figure S5.

Support from training programme directors was universally praised:

*‘We have very active, visible, approachable and fair-minded* [training programme directors]; *would be happy to seek advice*.’ (GP 8)

However, appreciation of deanery support was less consistent:

’*Deanery good at identifying issues, not so good at solving*.’ (GP 24)

Opinions varied on the Trainer’s Conference:

’*The regional conference is well organised and it is good to meet and network with trainers outside my local area*.’ (GP 2)

’*The Trainers Conference would be improved by more interactive small group work and less lectures*.’ (GP 49)

Recent restrictions in venues for development sessions, due to cost saving, was an issue for ESs.

Specific support suggestions called for:

’*Provision of a pool of tutorial resources*.’ (GP 58)

’*IT skills and organisational tips for GP trainers*.’(GP 48)

Better notice of events and more exam guidance was requested, in particular, advice on the ePortfolio and managing a difficult trainee:

’[The induction course should include the] *nuts and bolts of training*.’ (GP 24)

Online resources should have a well designed webpage with easy access to:

’*Details on* [for example] *BLS rules, child protection requirements, details of what is needed for ESR*.’ (GP 45)

’*Induction suggestions, trainee visit policy, data protection policy*.’ (GP 105)

’*Shared guidance/policies relevant to re-accrediting practices*.’ (GP 104)

More webinars and podcasts were welcomed.

## Discussion

### Summary

GP ESs remain a committed group of doctors, whose talents and successes should be celebrated.^14,15^ However, competition between training and clinical demands, an increased educational burden, and more trainees with variable needs have been identified in this study, contributing to statements that the next generation of GPs are less interested in becoming trainers. This corroborates other opinions^14,15^ that we may have reached a tipping point in the willingness of GPs to become trainers. Suggestions included consideration of the health of the supervisory relationship and extra supports for trainees with additional need.

### Strengths and limitations

The response rate was acceptable, as the average response for postgraduate faculty staff is 20%.^20,21^ While non-responders are usually less motivated and have lower morale than responders,^21,22^ unhappy supervisors may have taken this opportunity to record their frustrations, evidenced by the large contribution of free text data. This survey was carried out in only one region, a national survey may have been more representative. As this cohort of ESs operate within standard national structures and funding, it is likely that the results of this study are applicable to all UK trainers.

### Comparison with existing literature

Tensions between clinical demand and educational requirements are common in clinical teaching environments.^11,23^ Educator burnout is now worse among GP trainers than secondary care trainers, and has been increasing since 2018.^24^ Younger GP principals are less interested in becoming GP trainers than the previous generation,^15^ for reasons shown in this study.

ESs recognised the importance of workplace based assessments (WBAs), but similar to other studies,^25^ some view WBAs to be of low value and to detract from the quality of training. A meaningful educational alliance^26^ balances the supervisor’s dualistic role of trainee development and assessment.^8,10,13,26–28^ In designing WBAs, user–tool–context interactions should be considered, with WBAs nourishing the supervisory relationship.^27^ An excessive workload of assessment documentation is damaging to the supervisory relationship,^8,28^ in this study, only 18% of ESs had sufficient protected time for the portfolio. Future reviews of GP training WBAs may benefit from a focus on assessment of learning rather than performance,^28,29^ and promoting quality feedback as the linchpin of supervision.^26–28^

In GP training, there is adjacency to a senior practitioner, role modelling, and progress to legitimate participation consistent with the community of practice educational theory.^30,31^ With less fragmentation of training (less shift work) and more continuity of supervision than in other disciplines,^23^ this is protective of the trainees. But insufficient time for the needs of the trainee is worrying. This is especially in regards to the trainee in difficulty, whose number is increasing.^15,32^ ESs are good at identifying trainees with additional needs,^32^ but the extra burden for the trainer needs to be acknowledged and supported.

### Implications for research and practice

Research on how to develop faculty in postgraduate medical training of all disciplines is sparse, making this a promising area of future focus. Ideas were presented here about how to improve support of the ES. Recruitment into GP training may need to guarantee a basic minimum doctor competence. As recommended elsewhere,^15^ trainer induction courses need to be tailored to the work. Technology-enhanced learning can deliver websites, webinars, and podcasts in areas of need, and there may be future artificial intelligence applications that will help reduce the burden of training.^33^ Focused interventions may be useful, for example deanery staff checking in with an ES known to be challenged. There were lessons for the deanery, the Royal College of General Practitioners, and the General Medical Council, including consideration of supervisory relationship and the documentation burden.

## Data

The datasets (GP Educational Supervisor Survey results and the line by line coding) are available in the supplementary information.
